# Perceptions About Availability and Adequacy of Drinking Water in a Large California School District

**Published:** 2010-02-15

**Authors:** Anisha I. Patel, Laura M. Bogart, Mark A. Schuster, Kimberly E. Uyeda, Alexa Rabin

**Affiliations:** Department of Pediatrics and Institute for Health Policy Studies, University of California at San Francisco; Children’s Hospital Boston, Boston, Massachusetts; Children’s Hospital Boston, Boston, Massachusetts; Los Angeles Unified School District, Los Angeles, California; Alliant International University, San Diego, California

## Abstract

**Introduction:**

Concerns about the influence of sugar-sweetened beverage consumption on obesity have led experts to recommend that water be freely available in schools. We explored perceptions about the adequacy of drinking water provision in a large California school district to develop policies and programs to encourage student water consumption.

**Methods:**

From March to September 2007, we used semistructured interviews to ask 26 California key stakeholders — including school administrators and staff, health and nutrition agency representatives, and families — about school drinking water accessibility; attitudes about, facilitators of, and barriers to drinking water provision; and ideas for increasing water consumption. Interviews were analyzed to determine common themes.

**Results:**

Although stakeholders said that water was available from school drinking fountains, they expressed concerns about the appeal, taste, appearance, and safety of fountain water and worried about the affordability and environmental effect of bottled water sold in schools. Stakeholders supported efforts to improve free drinking water availability in schools, but perceived barriers (eg, cost) and mistaken beliefs that regulations and beverage contracts prohibit serving free water may prevent schools from doing so. Some schools provide water through cold-filtered water dispensers and self-serve water coolers.

**Conclusion:**

This is the first study to explore stakeholder perceptions about the adequacy of drinking water in US schools. Although limited in scope, our study suggests that water available in at least some schools may be inadequate. Collaborative efforts among schools, communities, and policy makers are needed to improve school drinking water provision.

## Introduction

From 1963 to 2004, obesity prevalence quadrupled among children and adolescents aged 6 to 19 years (1,2). Sugar-sweetened beverage consumption is linked to obesity (3), and the provision of water can reduce sugar-sweetened beverage intake among children and adolescents (4,5).

Recognizing the negative health effects of sugar-sweetened beverages, in 2004 the American Academy of Pediatrics stated that only water, real fruit and vegetable juices, and low-fat plain or flavored milk should be available in schools (6). In 2007 the Institute of Medicine recommended that all students have access to free drinking water (7).

Many US schools offer drinking water through 2 main sources: at no cost through drinking fountains and for purchase through vending machines and school stores. Water is not offered through the National School Lunch Program (NSLP), a US Department of Agriculture (USDA) program in which students receive nutritionally balanced breakfast and lunch at reduced or no cost (8).

Drinking fountains are the primary source of free drinking water in US schools, and state education departments set fountain maintenance requirements and dictate the number of fountains per student (9). However, few peer-reviewed studies have explored the adequacy of drinking water in US schools, and none have examined community members' or school officials' perceptions of school drinking water (10-14). A qualitative report, in which students photographed conditions in 4 urban US high schools, suggested that some schools may not meet minimum fountain-to-student ratios and that some may have nonfunctional fountains (10). Additionally, although local water utilities are responsible for ensuring that water meets state and federal drinking water standards, contaminants may enter water as it travels to buildings or through the corrosion of pipes, fixtures, and solder (15). Instead of replacing old pipes, many schools flush fountains at the start of the school day to decrease contaminant levels (16).

We investigated the availability and adequacy of drinking water in schools; attitudes about, barriers to, and facilitators of providing drinking water in schools; and ideas for increasing water consumption among students. We conducted semistructured interviews with key stakeholders (ie, school administrators and staff, public health and nutrition agency representatives, and families) in a large California school district to explore these issues.

## Methods

### Research context

Our research was conducted as part of a larger intervention that addressed disparities in obesity among middle school students in the Los Angeles Unified School District (LAUSD), the second-largest school district in the United States. During formative research we conducted (17,18), students and parents made unprompted, spontaneous requests for free, palatable drinking water in schools, and community stakeholders (eg, members of a nutrition advocacy organization, members of the school board) requested that our intervention address this need. When we tried to offer free, palatable drinking water in a school cafeteria as a part of the intervention, school officials with whom we met perceived logistical and regulatory barriers (eg, cost, beverage contract restrictions or federal regulations prohibiting offering water next to milk in cafeterias) to doing so. Therefore, we conducted this study to further examine the adequacy and availability of drinking water and barriers to provision of drinking water in schools to help develop interventions and policies to promote drinking water availability and consumption among students.

We interviewed key stakeholders from 4 California Unified School Districts: Los Angeles, Berkeley, Oakland, and Montebello. Because a study goal was to develop an intervention to increase drinking water provision in a LAUSD middle school, 16 of the 26 stakeholders were from LAUSD. Seventy-three percent of students from the LAUSD are Hispanic, 68% are eligible for free and reduced-price lunches through the NSLP (a marker for low household income), and 35% are classified as English learners (19). One stakeholder from each of the 3 other school districts was contacted to elaborate on innovative water programs that exist in their district schools.

The school board of LAUSD, the main district represented in our study, has worked to pass nutrition policy to address obesity among its nearly 700,000 students. The 2002 LAUSD Motion to Promote Healthy Beverage Sales banned most high-sugar beverage sales in schools and increased offerings of healthful beverages sold in schools (eg, bottled water) (20). Profits from beverage sales decreased until PepsiCo offered LAUSD a $1.81 million annual revenue enhancement contribution.

### Study design and sample

We conducted semistructured interviews with 26 key stakeholders from March to September of 2007. Participants were school and district administrators, school staff, public health and nutrition agency representatives, students, and parents. The initial 3 interviewees for this study were members of a community advisory board that informs our research center (University of California Los Angeles [UCLA]/RAND Center for Adolescent Health Promotion) about adolescent and family research priorities in Los Angeles. We used snowball sampling to obtain the remaining study sample (21) and analyzed interviews as they were conducted for preliminary themes. Participants were enrolled until theoretical saturation (ie, no new concepts were elicited) was reached (22). Verbal parental consent was obtained for the 2 students in the sample. The study was approved by RAND's Human Subjects Protection Committee.

Interviews were audiotaped and were approximately 60 minutes long. Interviews began with general questions about the participant's role in and perceptions of school food programs. Interviews continued with questions about school drinking water accessibility; attitudes about, barriers to, and facilitators of school drinking water provision; and ideas for interventions to improve drinking water availability. To validate some of the information given by participants and to obtain more specific information, we searched literature and the Internet, conducted school facility observations, and contacted additional potential interviewees not included in the initial sampling strategy.

### Data analysis

Content analysis, in which researchers develop codes, apply codes systematically to transcripts, and statistically test the reliability of multiple coders, was used to analyze data (23). Specifically, we used inductive coding techniques to develop a codebook that contained mutually exclusive themes (eg, perceived barriers to drinking water provision in schools) and subthemes (eg, cost of equipment and supplies for increasing drinking water availability). ATLAS.ti 5.0 qualitative data analysis software (ATLAS.ti Scientific Software Development GmbH, Berlin, Germany) was used to code, organize, and retrieve transcripts. Two research team members independently read transcripts, abstracted relevant quotes, and assigned codes to the quotes. Differences in code assignment were resolved through discussion. Cohen's κ was calculated to assess interrater reliability between coders for the 8 themes that emerged.

## Results

### Sample characteristics

A total of 880 coded quotes were obtained. Interrater reliability indicated excellent consistency between coders (mean κ = 0.99, range = 0.98-1.0) (24). Study participants were classified into 4 categories: district and school administrators (n = 8), school staff members (n = 7), nutrition and health agency representatives (n = 7), and families (n = 4, 2 students and 2 parents).

### Perceived problems with drinking water in schools

A common problem described by participants was the negative perception of tap water (Table 1). Many stakeholders stated that students did not drink tap water from school drinking fountains because of water safety concerns. Some stakeholders felt that family members' negative perceptions about tap water may influence students' decisions to drink tap water. On the basis of previous research, immigrant status was thought to contribute to such perceptions (25); many California schools have large numbers of students who emigrate from Latin American countries (eg, Mexico) where tap water is unsafe to drink and where bottled water consumption is among the highest worldwide (26).

Some administrators and teachers also expressed concern about the safety of water from school faucets and fountains. Many school employees said they did not drink from water fountains and encouraged their children to avoid tap water at school. Despite perceptions that school tap water is unsafe, 2 water quality employees stressed that tap water in schools undergoes strict testing and monitoring. A school district environmental health and safety employee said that school staff members flush drinking fountains at the start of the day to reduce levels of contaminants that can accumulate when water has been stagnant in pipes after periods of nonuse.

Another commonly mentioned concern about school drinking water was the poor taste and appearance of the water from drinking fountains. Fountain water was described by members of every stakeholder category as discolored, warm, and unpalatable. Participants also thought that school drinking fountains were old and outdated, as evidenced by the visible dirt, gum, or trash present. A few participants had concerns that drinking fountains were nonfunctional or were too few in number to meet student needs. Participants said drinking fountains may not accommodate as many students as possible (eg, not available near school athletic fields or where temporary trailers are installed). Additionally, some fountains are turned off, or water pressure is lowered to prevent students slipping on spilled water.

Some participants said that bottled water available for purchase in schools is too expensive. Despite such concerns, participants indicated that there was a demand for bottled water in schools. One principal noted that students still buy water and other beverages at school, even in a district where most students are from low-income backgrounds. Participants also mentioned concerns about the environmental effect of bottled water sales in schools. They worried that bottles were discarded in trash cans rather than recycled. Through observations and participants' reports we discovered that, although most schools did not have recycling programs, some school staff recycled items themselves.

### Perceived barriers to improving drinking water provision in schools

Cost was a frequently mentioned barrier to improving drinking water provision in schools (Table 2). Costs included labor and equipment for updating and maintaining existing fountains and introducing new programs (eg, serving water in pitchers at lunch, installing filters on fountains).

School officials said it would cost a tremendous amount to update fountains in schools. According to a news interview conducted with a local television station, 1 district superintendent said that it would cost approximately $53 million to install filters to remove lead in drinking fountains and $300 million to replace old lead pipes (27). School district administrators expressed concerns about costs for additional staff that would be needed to serve drinking water to students. Some participants stressed that maintaining existing facilities is a challenge because many schools have a backlog of deferred maintenance projects.

An additional barrier to providing drinking water in schools was participants' concern that beverage contracts prevent schools from offering drinking water other than through fountains or specified vendors. We did not discover any restrictions for providing free tap water to students in beverage contracts we explored, although some contracts prohibit serving free bottled water of a different brand than is specified in district contracts.

Another commonly mentioned barrier to improving the provision of drinking water in schools was the fear that serving drinking water to students may decrease the sales of competitive beverages (beverages sold separately from NSLP) that often fund school extracurricular activities. The 2 school fiscal managers interviewed said that their school profits from beverage sales range from $30,000 to $40,000 annually. If students drink free water served at school instead of purchasing competitive beverages that fund extracurricular activities, schools may have to seek alternative fund raising strategies.

Some participants believed that USDA regulations prohibited water from being served next to milk in school cafeterias. Indeed, 2 of 5 food service employees interviewed thought that schools that served water next to milk would not receive USDA reimbursement for school lunches. When we contacted USDA, we discovered that no restriction to prevent serving water in school cafeterias exists.

A few participants worried that if palatable water were available in school cafeterias, students might drink less milk. These participants mentioned that dairy consumption, which provides the calcium necessary for bone development (28), has decreased among students because of increased sugar-sweetened beverage consumption. However, most participants, who did not express such concerns, said that students were drinking sufficient quantities of milk outside of school and that students would continue to drink milk if water were served at meals. Other participants stated that water is a healthful alternative for students who have lactose intolerance or a milk protein allergy.

### Ways to improve the provision of drinking water in schools

Participants described existing programs to improve the provision of school drinking water. Some schools used funds from the Nutrition Network (a collaborative effort of local, state, and national partners to promote increased fruit and vegetable consumption and physical activity among low-income Californians) to purchase reusable water bottles for students to carry water with them at school. One parent sought funding from the city council to install a refrigerated filtered drinking fountain at his children's school. Teachers used personal funds to buy classroom water coolers. One California school district used district funds to provide water to all students daily by placing 5-gallon dispensers of cold, filtered water and paper cups in cafeteria settings. Other schools provided free bottled water for students in the cafeteria.

Participants had ideas for providing drinking water in schools. One participant proposed a water station where students could access cold water in a designated cafeteria area. One student requested that school vending machines dispense free water. Electric water coolers and filtered, refrigerated drinking fountains were also mentioned as options. Another common idea was to provide individual-sized bottles of water to students with cafeteria meals. Participants also offered ideas for offsetting costs. Schools with existing water programs used federal or district nutrition program funds, sought donations, or garnered parent and community support. Potential solutions posed by participants to decrease costs included obtaining bulk discounts for supplies, requiring that the USDA provide funds for drinking water at mealtimes, seeking foundation grants, and partnering with private corporations.

## Discussion

Decreasing sugar-sweetened beverage consumption is an obesity prevention strategy. Although schools are taking steps to increase offerings of healthful beverages sold in school stores and vending machines, free, safe, palatable drinking water may be lacking in schools. This study is the first to explore stakeholder perceptions regarding drinking water provision in US schools.

Consistent with prior research, a theme from this study was the concern regarding tap water safety (29). Another finding was worry about the appeal, taste, and appearance of water from school drinking fountains. Unlike previous research that examined drinking water provision in British schools (30,31), participants in this study infrequently mentioned concerns about the number of functioning fountains in schools. Although in a previous study, Irish schoolteachers perceived class disruptions (eg, water spills from reusable water bottles used in class, bathroom breaks) as primary barriers to increasing school water provision and consumption (32), such barriers were not predominant themes in our study.

Despite perceived barriers, participants had ideas for offsetting costs, and some schools already had water programs in place. In 2008, LAUSD instituted a program to test lead levels of drinking water sources from all district schools and to eliminate lead contamination in cases where lead levels were higher than the Environmental Protection Agency's action limit. Although we focused on California, other states have also established water programs in schools. One private corporation donated drinking fountain filters to 750 Utah schools (33). In New York City, the Department of Health and Mental Hygiene, in collaboration with the Department of Education, has installed water jets (large, clear plastic jugs that have a push lever for dispensing water) in some school cafeteria lunch lines (S. Baronberg, MPH, oral communication, October 2008).

Policy makers have also introduced legislation to increase the availability of clean, palatable drinking water in schools. California Assembly Bill no. 2704, legislation that sought to educate the public that beverage contracts and federal regulations do not prevent schools from offering students free tap water in cafeterias, was introduced in response to our research study's findings. Although this legislation was vetoed in 2008, introduction of this bill has opened the door for additional legislation (34). In his veto message Governor Schwarzenegger expressed interest in working with legislators on ways to promote the availability and consumption of clean water in California schools.

Legislators in other states have also introduced measures to increase the availability of clean, palatable school drinking water. In 2006, legislation was introduced in the US Senate that requires child care facilities outside of the home to be free from lead paint and lead-contaminated tap water in 5 years (35). In 2007, Senate legislation was introduced that mandates annual testing of school tap water, publishes tap water contamination reports, and eliminates any source of contamination (36).

### Study limitations

Our study has limitations. Although the qualitative methods we used allowed us to generate hypotheses and explore the issue of school drinking water provision in great depth and detail, our results are not meant to be generalizable. Future studies conducted with a representative sample of schools would help expand on the themes found in our study.

### Program and policy implications

Our results suggest that drinking water provision in some schools may be inadequate. Schools, communities, and policy makers should collaborate to develop programs and policies to ensure that free, clean, palatable drinking water is available to students. For example, to address concerns about tap water safety, schools may consider testing drinking fountain water for lead and other contaminants and educating school staff members, students, and parents about results. To counter misperceptions that USDA regulations and beverage contracts prohibit serving free tap water in school cafeterias, state education departments and school districts can clarify the absence of such restrictions. Given their financial and material resource limitations, schools may approach the private sector for funding or supplies (eg, reusable water bottles, filters, fountains) and consider environmentally friendly approaches (eg, biodegradable or recyclable cups, tap water rather than bottled water) to increase school water provision.

## Figures and Tables

**Table 1:** Perceived Problems With Drinking Water in Schools Among 26 Key Stakeholders, California, 2007

**Themes (No. of Participants Who Reported Theme)**	Participant Quotations
**Inadequacies of school drinking fountains**	
Poor taste and appearance of drinking fountain water (n = 17)	"Unfortunately, when kids go to the tap or fountains to drink water initially, the water may be warm or the water may be discolored." (school/district administrator)" [Fountain water] tastes a bit like tar." (student)
Fountains visually unappealing (n = 14)	"Ground workers do clean [the fountains] often, but still you have the little ones that come and do things they are not supposed to and it's considered dirty." (school staff member)" I know our facilities are neglected at these schools. There are kids who spit in the bubbler and leave their gum in there. So it can kind of be a gross, uninviting context for kids to drink out of." (school/district administrator)
Fountains nonfunctional or inadequate in number (n = 6)	"Now when I'm looking across here, most of our schools are reporting that their fountains are all clean and functioning. But that's not what we found." (nutrition and health agency representative) "We would start getting complaints that the drinking fountains weren't working. When we went out there it's because they'd turn them off to keep kids from playing with the water." (school/district administrator)
**Concerns about tap water**	
Negative public perceptions of tap water (n = 19)	"Parents always say, 'Oh, don't drink the water because that is nasty. . . . Oh no, just wait and buy a water bottle instead of drinking from the fountains.'" (student)" We're afraid of the water; we are doubting that the water is actually clean. And sometimes the water fountains, I think people think it's not clean, and [the] water is not clean." (nutrition and health agency representative)
Personal concern about tap water safety (n = 7)	"[My daughter] doesn't want to drink from the faucets. It's not clean; it's not, you know." (parent)" My kids that go to school, I don't like them drinking water from the faucet. I just think it's dirty. I think it's dirty, [there are] chemicals in it." (school staff member)
**Problems with bottled water sales in schools**	
Sale of bottled water in schools is harmful to the environment (n = 10)	"There's a lot of talk about the plastic bottles being a big environmental hazard. I think it's good if you could convince the kids that the water out of the drinking fountains is clean and healthy." (parent) "One of the biggest ecological problems today is those drinking water bottles, so for schools to perpetuate that in my mind would only make it worse." (nutrition and health agency representative)
Bottled water sold in schools is expensive (n = 9)	"All students are not fortunate to have money to buy bottled water." (school/district administrator) "Sometimes we don't take money to school, and how could we buy [bottled water] when we don't have money?" (student)

**Table 2:** Perceived Barriers to Improving Drinking Water Provision in Schools Among 26 Key Stakeholders, California, 2007

**Themes (No. of Participants Who Reported Theme)**	Participant Quotations
Cost of equipment and supplies (n = 19)	"School buildings are generally aging, and school budgets do not have the funding to keep up the maintenance, both with the cafeteria and the water fountains. So I do think that is an issue in many schools." (nutrition and health agency representative) "To put a drinking fountain in every classroom is a big cost. So the cost actually would be the biggest barrier." (parent) "I think the only barriers are financial." (school/district administrator) "If a company would donate it, water could be provided in schools more easily." (school staff member)
Beverage contract restrictions (n = 12)	"Local school districts have contracts that provide water, so you have to investigate whether providing water will go against these contracts or not. If there is a contract, one should go to the board to advocate for changes in future contracts." (nutrition and health agency representative) "Our only contract here is with Pepsi. I'm not really sure [if free water can be served in cafeterias]. I don't think so, no." (parent) "It is entirely possible that we have entered into contracts with vendors saying that they can sell water and other things at the student store, but we're not allowed to provide free water in the cafeteria." (school/district administrator) "[Serving free water in cafeterias] would be a conflict because that's by the student store . . . and there'd be competition for people drinking less of theirs, maybe drinking more of the one that's offered." (school staff member)
Decreased revenue from competitive beverages (n = 10)	"[Serving free water in schools is] an issue, but there is a list of 500 other ways to make money in the schools." (nutrition and health agency representative) "The sales would go down if something's free. The student store makes it so that we don't have to have fundraisers for the kids because, believe me, I'm not excited about running a fundraiser." (school/district administrator) "As for me [serving free water in schools] would hurt our water sales. Like I said, our water sales aren't great, but we have to rely on every sale we get right now because we don't generate the volume we used to." (school staff member) "Well, the school would lose money first of all, and the principal and the teachers would get upset because they're losing money, and they won't have enough money to have a field trip or for things that they use." (student)
US Department of Agriculture regulations prevent water being served next to milk (n = 6)	"In the eating areas there is nothing to prevent serving water there, but in the food service line it is still unclear." (nutrition and health agency representative)" There are a lot of misunderstandings among school food service directors, cafeteria workers, and even state employees, and I suspect probably federal government employees, that water is not allowed to be served anywhere in the cafeteria because the assumption is it might be in competition to buy a meal." (nutrition and health agency representative) "I probably should have a better understanding of these regulations, but the National School Lunch Program does not provide for water. It's not one of the reimbursable components. There are limitations on what can be sold in competition with the National School Lunch Program." (school/district administrator) "Wherever a reimbursable meal is offered, you have to offer milk as the beverage, and you can't confuse anybody by putting water in the milk cooler. So, you know, if we have an audit, the auditor can't think that the water comes with the lunch." (school staff member)
Concerns about decreased milk consumption (n = 4)	"I do think that to have water right next to milk, you do undermine slightly the milk. There'd be some kids that just, you know, opt for the water. Milk consumption is way down so we really would like to encourage milk consumption." (nutrition and health agency representative) "Water should be available as a milk replacement in schools. My kids have milk at breakfast and dinnertime; they don't need it at lunch. Some feel that a lot of nutrients are delivered via milk; figuring this out is challenging." (nutrition and health agency representative) "If we offered [water], the kids wouldn't drink the milk. And you know they need 3 servings of calcium a day; you know some in their teenage years need more than that." (school staff member) "Well, water's good for you, but I think milk's healthier for you. I mean, a lot of kids here actually drink milk." (school staff member)
